# Reverse Alphabetical Grafting: An Innovation to Ensure Successful Split-Thickness Skin Grafting

**DOI:** 10.1097/GOX.0000000000002317

**Published:** 2019-07-05

**Authors:** Wan Xian Esther Tan, Jing Tzer Lee, Gavin Chun Wui Kang, Gale Jue Shuang Lim

**Affiliations:** From the * KK Women’s and Children’s Hospital; † National University Hospital, Singapore.

The key to successful skin grafting requires optimization of 3 major components^1^:

1. Preoperative patient factors: nutrition and wound bed preparation;
2. Surgical factors: good harvesting technique, stabilization of the graft to avoid shearing, and adequate hemostasis of the wound bed to avoid hematoma formation; and
3. Postoperative wound care: skin graft anchored to ensure stability.

For the survival of the skin graft, the dermal side needs to be placed against the wound bed^2^; one of the reasons for non-take of a skin graft is due to incorrect application onto the wound bed, that is, the epidermis-side onto the wound bed.^3^ Unlike adults, a child’s skin is fairer, smoother, thinner,^4^ and has a paucity of hair follicles. This makes it difficult to differentiate the epidermis from the dermis, thus increasing the risk of incorrect placement. This is associated with morbidities such as the need for repeated procedures under general anesthesia, additional donor site morbidity, prolonged dressing, increased cost, and unhappy parents.

**Fig. 1.**
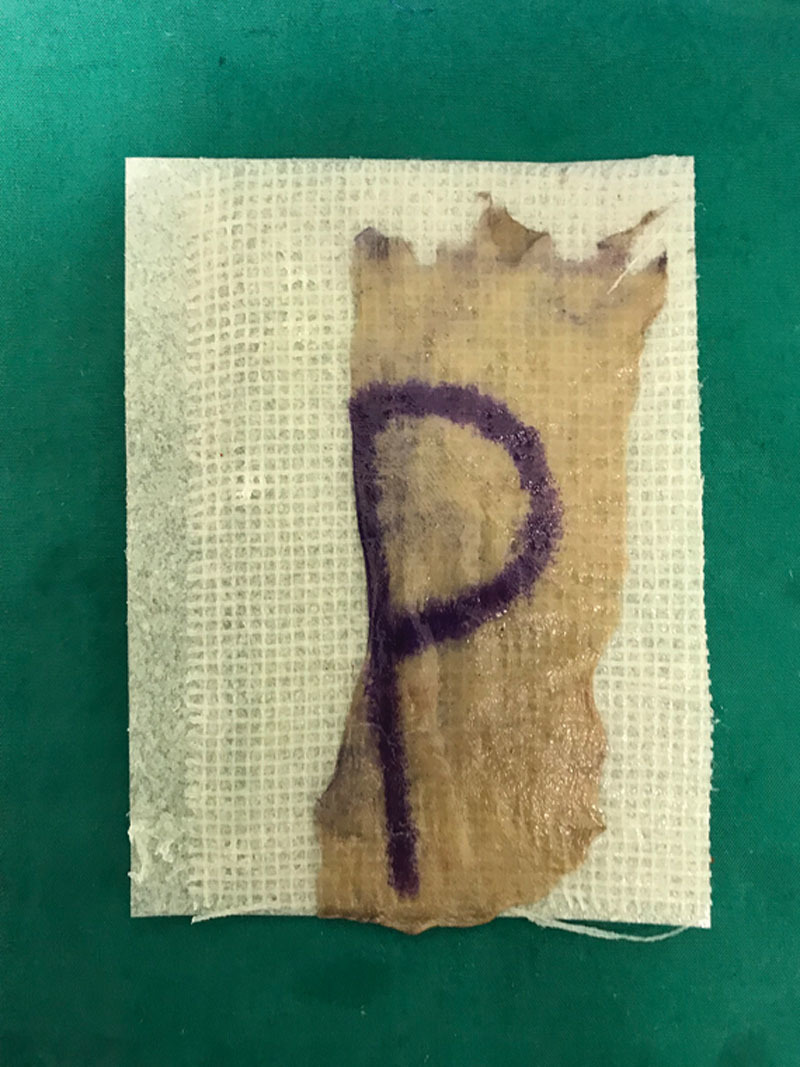
Skin graft with epidermis side facing upward, marked with “p.”

**Fig. 2.**
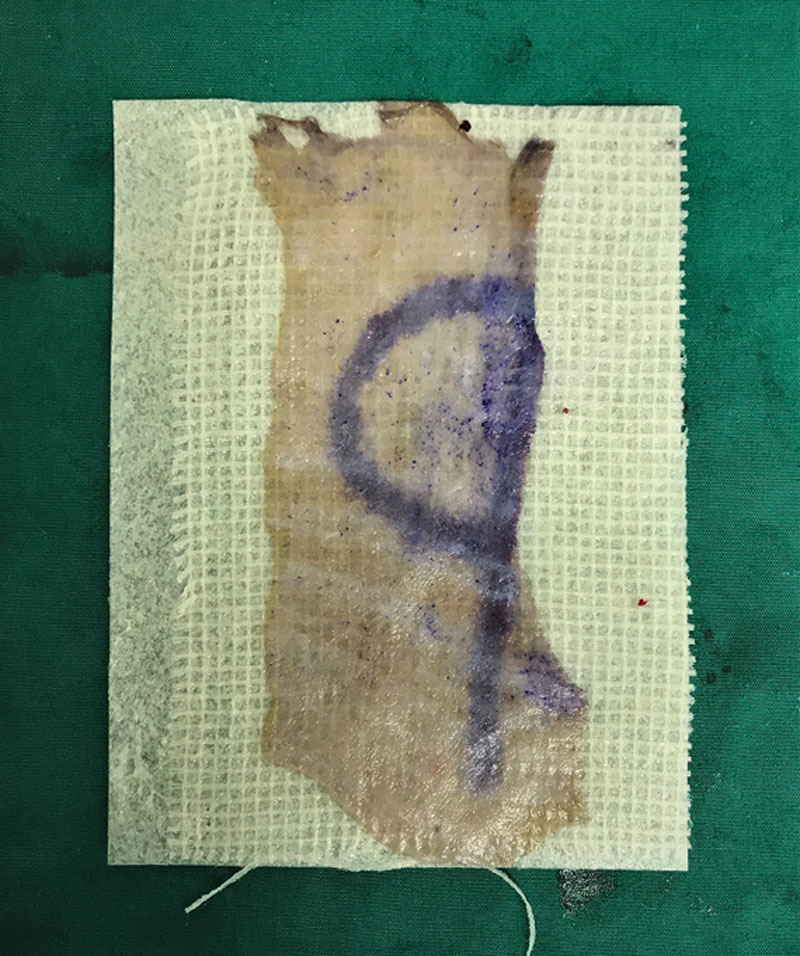
Skin graft with dermis side facing upwards, “p” shown as “q.”

At our center, the KK Women’s and Children’s Hospital in Singapore, we describe a simple and novel technique to identify the dermal side of the harvested graft to ensure safe placement during split-thickness skin grafting. To aid the identification of epidermis and dermis, a letter of the alphabet is drawn on the epidermis before harvesting. The markings are made using a sterile skin marker or methylene blue dye that will fade postoperatively. As shown in the picture, the letter “p” is marked out on the epidermis. The skin graft is then harvested using a powered dermatome with an adjustable guard set to a variable thickness in accordance with the age of the child and the donor site selected. On the freed graft, the side with the letter “p” suggests the epidermal side (Figure 1), whereas a flipped-over “q” would suggest the dermal side (Figure 2). In correct placement, the operator should see a “p.” On the other hand, if a “q” is seen, it would suggest incorrect placement. Other asymmetrical alphabets can be used to aid identification, for example, “b” which would read as “d” on the dermal surface. Conversely, alphabets that are symmetrical such as “w” and “o” are unsuitable as they would look the same when flipped over.

When indicated, meshing or fenestration is then performed on the skin graft without changing its orientation.^5^ The skin graft (meshed or unmeshed) is then laid out on a piece of tulle gras. Placement on the tulle gras may cause the skin graft to be shiny on both sides, along with the paraffin oil used for harvesting the skin graft, increasing the difficulty of differentiating between dermal or epidermal side. With the epidermis side in direct contact with the tulle gras, the reversed alphabet will show up clearly through the dermis, which is facing the operator, and is ready for a safe application.

The advantages of RAG are:

1. Safe—minimizes the risk of misplacement of graft.
2. Simple—easily reproducible for multiple strips of skin graft or for technically challenging cases, especially in younger children. This uncomplicated step can be carried out by all operators regardless of the level of experience. This can also reduce confusion in a surgical team with multiple operators.
3. Decreases operating time by decreasing time taken to determine the correct side of skin graft.

In summary, with good preoperative optimization and postoperative care, RAG is a novel adjunct that involves the use of alphabets to facilitate accurate on-table judgment of skin graft surface orientation, a crucial step in the success of skin grafting.
